# Category Congruence of Display-Only Products Influences Attention and Purchase Decisions

**DOI:** 10.3389/fnins.2021.610060

**Published:** 2021-08-26

**Authors:** Uma R. Karmarkar, Ann L. Carroll, Marina Burke, Shori Hijikata

**Affiliations:** ^1^Rady School of Management, School of Global Policy and Strategy, University of California, San Diego, San Diego, CA, United States; ^2^Department of Psychology, Northwestern University, Evanston, IL, United States; ^3^Statistical Analysis System Institute, Cary, NC, United States; ^4^Antler, Sydney, NSW, Australia

**Keywords:** retail, e-commerce, attention, decision making, choice-behavior, consumer neuroscience, eyetracking, neuromarketing

## Abstract

In e-commerce settings, shoppers can navigate to product-specific pages on which they are asked to make yes-or-no decisions about buying a particular item. Beyond that target, there are often other products displayed on the page, such as those suggested by the retailers’ recommendation systems, that can influence consumers’ buying behavior. We propose that display items that come from the same category as the target product (matched) may enhance target purchase by increasing the attractiveness of the presented opportunity. Contrasting this, mismatched display items may reduce purchase by raising awareness of opportunity costs. Eye-tracking was used to explore this framework by examining how different types of displays influenced visual attention. Although target purchase rates were higher for products with matched vs. mismatched displays, there was no difference in fixation time for the target images. However, participants attended to mismatched display items for more time than they did for matched ones consistent with the hypothesized processes. In addition, increases in display attractiveness increased target purchase, but only for matched items, in line with supporting the target category. Given the importance of relative attention and information in determining the impact of display items, we replicated the overall purchase effect across varying amounts of available display information in a second behavioral study. This demonstration of robustness supports the translational relevance of these findings for application in industry.

## Introduction

The rise of e-commerce has expanded the scope of retail design and the range of consumer choice settings. As one example, a carefully planned brick and mortar grocery store layout or planogram creates specific product adjacencies that shoppers can become accustomed to, like knowing the eggs are “always” near the milk. However, the flexible nature of digital item displays online allows for rapid updating of the choice context across multiple stages of a shopper’s decision, with the possibility of viewing a particular product in settings that differ across pages designed for search, interest, selection, and purchase (see also Häubl and Trifts, 2000; Senecal et al., 2005).

Along similar lines, digital retail has created distinct and/or novel purchase decision steps and pathways. Generally speaking, models of the consumer decision-making process, or “decision funnels,” begin with generalized awareness of a product, perhaps as a member of a larger set of options (e.g., Gourville and Norton, 2014). They proceed to narrow, with some form of engagement or information-seeking about more constrained consideration sets, to evaluation of specific options leading to a purchase decision or decisions. When shopping online, consumers in the information seeking and evaluation stages can arrive at product pages that are designed around a single option. In this setting, consumers can evaluate the product, and are prompted to move forward in deciding whether or not to select it for purchase via buttons for “add to cart” or “buy now.”

Though the purpose of individual item pages is to offer more information about a specific product, it is common to have other information, including other products, visible on the screen. In many such settings the firm’s recommendation system might display a set of similar products within the same general category. Alternately, the retailer may cross-promote products from different categories that they (or their algorithms) believe will also appeal to the shopper. These display sets are not central to the consumer’s choice of whether (or not) they want to purchase the target, which is the primary purpose of the page. They are often intended to increase overall basket size by complementing or supplementing that decision, and can even appear “subsequent” to purchase or add-to-cart buttons, lower down on the page. However, they alter the choice context, and thus have the potential to influence the choice itself (e.g., Bettman et al., 1998).

Prior research using neuroscientific methods suggests that non-choice items do elicit decision-related evaluations, including representation of their value in areas such as ventral striatum and mPFC (e.g., Lebreton et al., 2009; Tusche et al., 2010; Levy et al., 2011; Smith et al., 2014). In addition, it has been shown that a network of areas including pregenual ACC and ventral striatum automatically encodes the average value of a set of items if they are viewed together, regardless of whether a person is just browsing or making an active choice (Shenhav and Karmarkar, 2019). These findings suggest that display items’ values are likely to be represented in corticostriatal reward-related circuitry even when a consumer is focused on the target. Given this evidence, it seems likely that shoppers attend to display items when present, however briefly, and that these products could be integrated into the decision process.

Behavioral studies involving attractive but unavailable “phantom” items, as well as product stockouts, have shown how non-choice items can influence decisions in various ways (e.g., Pratkanis and Farquhar, 1992; Farquhar and Pratkanis, 1993; Fitzsimons, 2000; Scarpi and Pizzi, 2013; Trueblood and Pettibone, 2017). The nature of these effects can depend on whether consumers learn that the option is unavailable before or after making their choice (Pratkanis and Farquhar, 1992; Scarpi and Pizzi, 2013). Fundamentally though, these non-choice items are offered to the consumer as meaningful elements in an active choice set and indeed are able to create the same kinds of biases that would be expected if they were still valid options under consideration (e.g., Doyle et al., 1999).

The display items presented on a target product’s e-commerce page are not necessarily unavailable, but they are “non-choice” because they are peripheral to the focal yes-or-no target purchase decision. Previous work has shown that they exert an influence that is dependent on their *similarity* to the target products (Karmarkar, 2017; Friedman et al., 2018). Karmarkar (2017) showed that purchase intent for a target item is increased when display and target items come from the same product category, and decreased when display and target items’ categories are mismatched. This effect is consistent with findings that high value phantom products enhance interest in the most similar options that are still available (e.g., Pettibone and Wedell, 2000). Notably, though, the relative value of the display items as compared to the target does not influence purchase (Karmarkar, 2017; Friedman et al., 2018). This lies in contradiction of many choice framing or decoy effects that arise from comparisons of value among options in a choice set (e.g., Huber et al., 1982; Simonson, 1989; see also Louie et al., 2013; Webb et al., 2021).

Overall, then, it does not appear that consumers are processing display items as active or valid choice options. Instead we propose that display items create choice frames or contexts that influence the attractiveness of the purchase decision rather than impacting the perceived value of the target. This distinction can be considered as analogous to the distinction between transaction and acquisition utility (e.g., Thaler, 1985). While the definition of transaction utility is based on price perceptions, we propose a similar type of context-dependent “category-based” transaction value mechanism created by congruency between the item and the display.

In particular, our framework suggests two contributing mechanisms. First, display-only items that match the target product category may serve to enhance purchase intent by raising the attractiveness or appeal of an in-category purchase. This predicts that increased attractiveness of the display items in the matched condition could increase purchase likelihood, which we test in different ways across Study 1 and 2. Second, prior work demonstrating that consumers often neglect opportunity costs when making yes or no choices (e.g., Northcraft and Neale, 1986; Jones et al., 1998; Frederick et al., 2009; Spiller, 2011; Greenberg and Spiller, 2016). In particular, such studies showed that reframing a choice from *buy/don’t buy* to *buy this product/keep the money for other products* reduces purchase intent by reminding people of other opportunities for those funds (e.g., Frederick et al., 2009). Mismatched display-only items offer a conceptual parallel to that manipulation by raising the salience of other item categories within the current decision context and engaging shoppers’ attention and processing related to those categories. While matched displays may frame a choice as “Given this category, do you want to buy this item,” mismatched displays may reframe it as “Given that you could spend money on any of these categories, do you want to buy this particular item?”

The proposed mechanisms offer complementary hypotheses related to information processing when considering a target product in the presence of other display-only items. First, given that increased attention is correlated with increased purchase intent (e.g., Krajbich et al., 2010; Krajbich et al., 2012), it may be that shoppers pay more attention to the target item in the matched condition than in the mismatched condition. Similar logic then suggests that shoppers may pay more attention to the display items in the mismatched condition than the matched condition. Specifically, if the mismatched display items are raising awareness of the value of other categories, they would be predicted to capture additional attention.

In Study 1, we use an eye-tracking experiment to examine these hypotheses directly using purchase decisions about a target product made in the presence of other product images. This design features a highly simplified version of the detailed information that consumers might face on a firm’s site. To help address this, in Study 2, we replicate the display-set effects in contexts that feature more information–price and product labels for the display items as well as the target items–speaking to the relevance of these findings for practical applications. Collectively this work offers an enhanced understanding of how non-choice product information, such as that generated by e-commerce recommendation systems, can influence interest and/or evaluation stages of the decision process in ways that impact subsequent purchase.

## Study 1

### Methods

The design of this study was approved by the Harvard Business School’s Internal Review Board. Sixty-six individuals under the age of 40 participated in the eye-tracking experiment. Fixation detection rate was measured as the Tobii Pro Studio “weighted gaze samples” statistic calculated by dividing the number of eyetracking samples that were correctly identified by the total number of samples. Eight of the participants had a fixation detection rate that fell below 50%, and were excluded from analysis, leaving a sample of 58 (*M*_Age_ = 24.43, *F* = 25). The study was conducted as an independent experiment in a single 30–45 min session that did not include other studies or surveys beyond those reported. All participants were informed that the study involved eye-tracking in the recruitment process, gave consent prior to engaging in study activities and were given additional opportunities to ask questions and/or withdraw consent after the complete task instructions were explained.

Participants viewed the tasks on a computer screen, using a keyboard to move through the instructions and a mouse to indicate their choices and ratings. Stimuli presentation and data collection were conducted using the Tobii Studio software (v3.4.3) and a Tobii X2-60 eye-tracker (1920 × 1080 recording resolution, 60 Hz sampling rate, 4° accuracy). After individual calibration of the eye-tracker, it was explained that participants would be asked to decide whether they wanted to purchase several different target products, but that additional products or images would be displayed on the screen. Targets were familiar consumer products selected based on an age demographic of 18–40 years old, drawn from a range of categories such as office supplies, gifts, apparel, and personal electronics. Participants were further informed that they would not be able to choose or receive any of the display-only images that they might see during the experiment.

**FIGURE 1:**
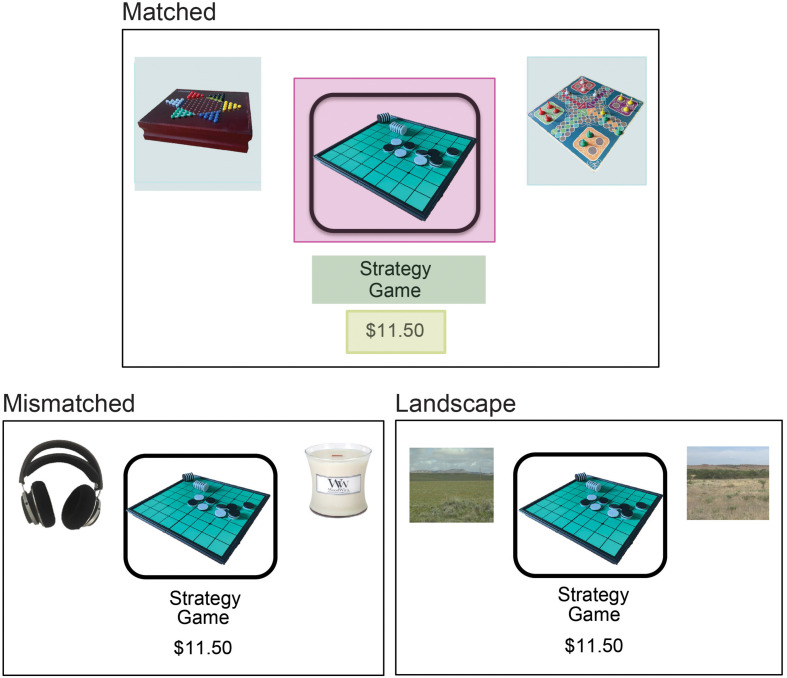
Study 1 sample presentation stimuli. Shaded areas in the Matched condition illustrate the nature of AOI placement for the target image, display images, target label, and target price. Images are for illustration purposes only. See experimental materials for exact stimuli.

On each of the 36 decision trials, a fixation cross was presented at the center of the top third of the screen for 1 s. The screen automatically advanced to show the main stimulus, in which the target product was presented in the center of the screen together with text indicating its name and its price (Figure 1). The target was flanked by two display-only images falling into one of three conditions: landscapes, category-matched products, and category-mismatched products (Figure 1). Landscape images were selected to avoid the presence of distinct landmarks or strong individually differentiating features. Each “product page” (stimulus presentation) was shown for 5 s. After this, the screen automatically advanced and participants used a computer mouse selected their willingness to buy the target from the following set of choices [Strong No, No, Weak No, Weak Yes, Yes, and Strong Yes] (as in Karmarkar, 2017). The rating period was self-paced; after participants selected their response they clicked on a “Finished” button to submit the answer, and the next trial automatically began.

An extended description of the stimuli and trial information can be found in the Supplementary Methods. Briefly, 36 products that were widely available for retail purchase and generally desirable for the 18–40 year old demographic were chosen as target items. Purchase trials across the landscape, matched and mismatched conditions were intermixed across conditions, although all participants saw the target items presented in the same order. Stimulus presentation across conditions was counterbalanced among participants, such that for each target item, approximately one third of the participants viewed it with matched display products, approximately one third viewed it with mismatched display products, and the rest viewed it with landscape images. This design was intended to prevent condition-specific effects from depending on (or correlating with) the nature or attributes of any particular target item or its category.

Visual areas of interests (AOIs) used for measuring the duration of fixation times were centered on product image (target, left display image, and right display image) as well as on the target product label text and target price text (see Figure 1 for illustration). The focal item presentation was standardized across all trials and categories with the item in a black outlined box at the center of the screen, with a rectangular AOI extending 50 px beyond this object (AOI: 171754 px). The landscape non-choice AOIs were all identically sized (103041 px), and represent the largest AOIs other than the target. This offers a conservative control for the other matched and mismatched conditions since the large landscape AOIs had the highest probability of capturing fixations. While display items varied in size, across all stimuli AOIs were non-overlapping and defined with a 50 pixel border beyond the underlying image/text boundaries. Fixations were classified with an I-VT filter (Tobii Pro Studio). Given varying rates of fixation detection across participants, fixation duration analyses reflect trials in which shoppers allocated a non-zero amount of attention to the AOIs studied.

Participants were endowed with $15 at the start of the experiment independently of the main study compensation, and their choices were incentive compatible. Participants learned that one of their decisions would be selected at random to count “for real” at the end of the study. If the participant had selected any of the three *no* answers, they retained the full endowment. If they had selected any of the three *yes* answers, they paid the listed item price from their endowment, retained any residual funds, and received the chosen product in the mail.

After the eye-tracking portion of the experiment, participants engaged in a behavioral rating survey (Qualtrics XM) for each of 108 products (36 targets, 24 matched display products, 24 mismatched display products, and 24 unseen display products). Landscape images were not included in the ratings. For each item, participants used a computer mouse to indicate whether they remembered viewing the item during the primary task on a four point scale labeled [Definitely No, Maybe No, Maybe Yes, and Definitely Yes] and then rating their liking for the item on a seven point scale ranging from Do Not Like to Like Very Much. Three participants did not have enough time during the experimental session to complete these ratings, resulting in a sample size of *n* = 55 for analyses related to liking and recall.

Statistical analyses (behavior, eye-tracking, and their combined relationships) were conducted by importing the aggregated data into Stata/SE software. Study materials and a link to the OSF repository of the individual eyetracking raw data files can be found here: https://researchbox.org/304.

### Results

While the six-point decision response scale was initially used to keep methods consistent with prior demonstrations of this effect (e.g., Karmarkar, 2017), for the purposes of incentive compatibility, purchase responses across the scale were collapsed into *yes* and *no*. This approach was also considered more representative of the yes/no choice that was explained to participants and that would be normally made on a retailer’s site. As a result, purchase data is modeled as a binary choice in the following analyses.

While a range of factors can influence purchasing decisions, there is reasonable agreement that such choices are likely to reflect individual preferences for the good, balanced with sensitivity to its price (see also Prelec and Loewenstein, 1998; Knutson et al., 2007; Karmarkar et al., 2015). Thus as a control for the overall quality of the decision process captured by this experiment, the binary purchase decision was modeled with a mixed-effect logistic regression on target liking and target price (Model Wald X^2^ = 300.89, *p* < 0.001). To represent the potential variance in individual purchasing likelihood, susceptibility to the effects, and the within-subjects design, the model included subject random effects (*n* = 55 groups, three participants did not complete the liking ratings). As might be expected, there was a significant positive coefficient on liking (*b* = 1.43, SE = 0.08, *p* < 0.01) and a negative overall effect of price (*b* = −0.12, SE = 0.03, *p* < 0.001). This analysis suggests that the choice process evoked by this task is consistent with meaningful considerations of costs and benefits as they might occur in real world purchasing. However, it is important to acknowledge that liking ratings were taken at the end of the study, and it is thus possible that they could have been influenced by the purchase decisions made during the task (e.g., Botti and Iyengar, 2004).

**FIGURE 2:**
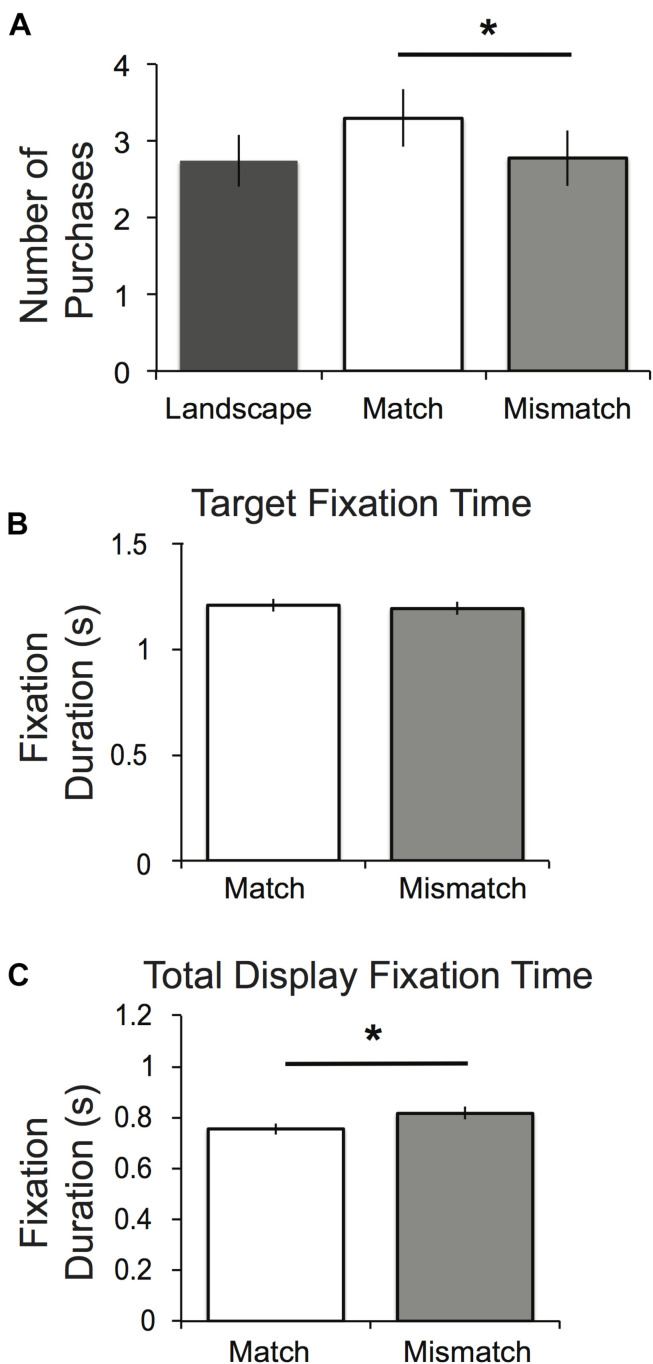
Study 1 results. **(A)** Number of purchases (incentive compatible “yes” decisions) made in each experimental condition. **(B)** Average of the total fixation time spent on the target image per trial in the matched (*M* = 1.21, SE = 0.0301) and mismatched (*M* = 1.19, SE = 0.0312) conditions. **(C)** Average of the total fixation time spent on the display images per trial in the matched (*M* = 0.755, SE = 0.0222) and mismatched (*M* = 0.816, SE = 0.0246) conditions. Error bars reflect standard error. *p < 0.05.

We then tested the impact of display on choice behavior across the three display conditions (landscape, matched and mismatched). This was done with a mixed-effects logistic regression with dummy variables for the matched and mismatched conditions (yielding the landscape condition as the reference), and subject random effects. As shown in Table 1, this analysis revealed a significant effect of the matched display (*b* = 0.313, SE = 0.140, *p* = 0.026), and no significant effects of the mismatched display (*b* = 0.02, SE = 0.143, *p* = 0.886; as compared to the landscape condition.) *Post hoc* comparisons of coefficients further demonstrated that purchase likelihood was higher in the matched condition compared to the mismatched one (*p* = 0.0365). Complementing this analysis, Figure 2A illustrates the average number of purchases, or “yes” responses that participants made during the twelve incentive compatible decisions in each condition. The increased purchase rate in the matched vs. mismatched condition is consistent with prior demonstrations of display-set effects (e.g., Karmarkar, 2017; Friedman et al., 2018).

**TABLE 1:** Mixed effects logistic regressions examining factors related to target purchase.

	Purchase	Purchase
Match	0.313* (0.140)	0.555** (0.179)
Mismatch	0.0206 (0.144)	0.281 (0.185)
Price		−0.0865** (0.0305)
Target Fixation Duration		0.389** (0.132)
Display Fixation Duration		−0.448** (0.169)
Model Wald X^2^	6.35*	33.42***
# obs	2088	1574
# groups	58	58

All models conducted with subject random effects. Standard errors in parentheses. *p < 0.05, **p < 0.01, ***p < 0.001.

A broader model of the impact of condition on purchase captured additional factors that might influence choice during the stimulus evaluation period. Specifically, it included target item price and variables for attention as measured by the total duration of fixation time (in ms) spent in the AOI centered on the image of the target product or the two display images (Note this influences the number of observations retained in the model, as fixations may not have been detected in all trials for all participants). As seen in Table 1, there was again a significant effect of the matched but not mismatched displays, accompanied by negative effects of price. In addition, consistent with the assumptions made in our framework, attention to the target item had a significant positive relationship with purchase likelihood, while attention to the display images had a significant negative relationship with target purchase.

Reflecting our specific hypotheses, we predict that attention to the target might be increased in the matched vs. mismatched conditions, reflecting the relationship between attention and purchase likelihood (e.g., Krajbich et al., 2010; Krajbich et al., 2012). Mixed effects regression on the fixation duration for the target revealed a significant negative effect for both the matched (*b* = −0.240, SE = 0.031, *p* < 0.001) and mismatched (*b* = −0.266, SE = 0.031, *p* < 0.001) displays (Table 2). Thus participants spent relatively less time attending to the target items when they were part of a multi-product display compared to when they were presented with landscape images. However, our *post hoc* comparison of coefficients found no significant difference between the times for the matched and mismatched conditions, suggesting that the category of the display products did not influence the amount of attention paid to the focal item (*p* = 0.406, mean fixation duration times shown in Figure 2B)^1^.

**TABLE 2:** Mixed effects regressions of total fixation times detected in target or display item AOIs.

	Target Fixation	Display Items Fixation
Matched	−0.240*** (0.031)	0.278*** (0.0290)
Mismatched	−0.266*** (0.031)	0.340*** (0.0293)
Model Wald X^2^	87.67***	147.39***
#obs	2006	1606
#groups	58	58

All models conducted with subject random effects. Standard errors in parentheses. ***p < 0.001.

A complementary hypothesis suggests that individuals attend more to the display items in the mismatched condition, suggesting an increased attention to outside options and opportunity costs, compared to the matched one. As shown in Table 2 (right column), mixed effects regressions showed that total fixation time (summed across the AOIs for the left and right display images) was increased for both the matched (*b* = 0.278, SE = 0.29, *p* < 0.001) and mismatched (*b* = 0.33, SE = 0.029, *p* < 0.001) products compared to the landscape images. In addition, a test for equality of coefficients revealed a significant difference for the mismatched display items such that they were attended to for longer (*p* < 0.023; mean fixation duration times shown in Figure 2C).

A few patterns emerge from this data. First, attention meaningfully differs between the landscape and the two product display conditions. Thus the presence of recognizable products appears to engage processes that go beyond those evoked by the mere presence of visual information unrelated to the target. Second, attention to the target item remains similar across matched and mismatched product displays, but the mismatched display items themselves capture more attention than the matched ones. This is consistent with our hypothesis that the mismatched display items decrease purchase of the target by highlighting the values and consideration of outside options and/or categories.

In addition to the absence of differences in fixation time on the target image, there was no significant effect of display condition on target recall or on target liking ratings. Specifically, regressing target liking on the condition dummy variables (with subject random effects) demonstrated no significant coefficient for the matched (*b* = 0.0744, SE = 0.098, *p* = 0.45) or mismatched (*b* = 0.012, SE = 0.098, *p* = 0.902) displays (with landscape again serving as the reference). Regressing target recall on condition (with subject random effects) also showed no significant effect for either matched or mismatched displays (match: *b* = 0.0227, SE = 0.0223, *p* = 0.307; mismatch: *b* = 0.018, SE = 0.0223, *p* = 0.414).

This suggests that the matched display items might exert an influence on the decision process overall rather than the target value in particular. Our framework proposes that matched display items are acting as supporting evidence for purchase within the target category, despite being unavailable themselves. If this were the case, more preferred display items would be expected to be more effective at increasing purchase likelihood for the target (see also Karmarkar, 2017), though the prediction for the mismatched condition is less clear.

We conducted a mixed effects logistic regression with subject random effects on the influence of display-item liking and condition (match vs. mismatch only) on the yes/no purchase decisions. Display-item liking was coded as the averaged liking ratings for the two display items presented in the trial. The model found a significant interaction between display-item-liking and condition (*b* = 0.49, SD = 0.100, *p* < 0.001). There was no main effect of display-item-liking (*b* = 0.017, SD = 0.071, *p* = 0.808), and a negative effect of the matched (vs. mismatched) condition (*b* = −1.78, SD = 0.44, *p* < 0.001). To interpret the interaction, we examined the impact of liking for the display items on purchase for the 12 trials in the mismatched and 12 trials in the matched conditions in separate models (Table 3). In the matched case, a mixed effects logistic regression showed that average display-item liking had a significant positive effect on purchase in line with our predictions (*p* < 0.001; number of observations reflects that one participant had one missing liking rating). However, the effects of display-item liking were not significant when modeled for the mismatched condition (*p* = 0.626). Again, this analysis is accompanied by the caveat that display liking ratings were conducted after the main choice task. Thus they may have been influenced by the display conditions and/or purchase decisions themselves.

**TABLE 3:** Mixed effects logistic regressions of purchase on liking ratings for the display items in the matched or mismatched trials.

	Matched Only Trials	Mismatched Only Trials
	Purchase	Purchase
Display Liking	0.581*** (0.081)	−0.0382 (0.078)
Model Wald X^2^	51.69***	0.24
#obs	659	660
#groups	55	55

All models conducted with subject random effects. Standard errors in parentheses. ***p < 0.001.

Collectively our results suggest that these purchase decisions rely on perceptions of the full set of visible items rather than the information specific to the target item. For example, target liking, target fixation, and target recall were similar across matched and mismatched conditions despite differences in purchase rates. Instead, the purchase-relevant differences that did arise were primarily related to the display items and their relationship to the target. This offers an interesting clarification on the role of the display, suggesting that its impact is on the decision process rather than focused on the decision target. This is further reinforced by the differences between the landscape trials and the trials where the “non-choice” images were of recognizable products.

While the sparse design of Study 1 allows for more precise inferences about attention, it does reflect an information asymmetry between the target and display options. Thus marketers may ask whether display-set effects are still expressed when the display items also bear descriptive labels and prices, or whether an increased availability (and thus increased salience) of display-item information might moderate the results. In addition, while Study 1 shows that that liking for the individual display products is correlated with purchase of the target, our framework also proposes that this increased liking increases the overall attractiveness of the “offer,” reflecting the complete set of items. Thus in Study 2, we test the robustness of the purchase effects across varying amounts of available information for the non-target products, and also examine the relationship between purchase likelihood and the value of the overall set.

## Study 2

### Methods

The research protocol was approved by the University of California, San Diego, Internal Review Board. Four hundred and fourteen individuals completed this study online for monetary compensation via the Amazon Mechanical Turk platform (*M*_Age_ = 37, *F* = 204). The reported sample reflects the exclusion of 69 participants who failed to correctly answer two comprehension questions (All participants received compensation, regardless of their performance). First, on the instruction screen, participants were asked to type in the words “I understand” to indicate their comprehension of the task instructions. Second, at the end of the survey participants were asked “Out of all of the items you saw in the survey, which one do you think you would be the most likely to buy if you were in a store?” Participants who left the question blank, or entered in inappropriate text (e.g., “GOOD” or “nice survey”) were qualified as exclusions.

All participants assented to participate following reading an information sheet, and then filled in a “captcha” image recognition puzzle (not used for exclusion). Participants were randomly assigned to one of six experimental conditions based on a 2 × 3 between-subject design that varied the categories of the display options (Matched vs. Mismatched) and the individual item information (Control, Label Only, and Label + Price). The stimuli in the control condition used the same presentation design as the matched and mismatched conditions in Study 1, in which a descriptive text label and price was only available for the target item. In the label condition, a descriptive text label was presented together with all three products, and no price information was listed. Finally, in the label + price condition, a descriptive text label and price was presented for all three of the visible products. All participants were asked to imagine that they were shopping for themselves online, and that they would see a display from an online store. They further learned they would be indicating whether they would buy the item specified in the center of the screen. Participants viewed the stimuli for 4 s, and then indicated their purchase intent on a six-point scale from Strong No to Strong Yes. Participants made decisions for three different items (three trials)–an art print, a game, and bath towels. These decisions were hypothetical, such that participants did not receive any of the items they viewed. Following the decision task, participants also rated the attractiveness of the display of items overall (comprised of target and display items) on a seven-point scale from Not at all Attractive to Very Attractive.

### Results

**FIGURE 3:**
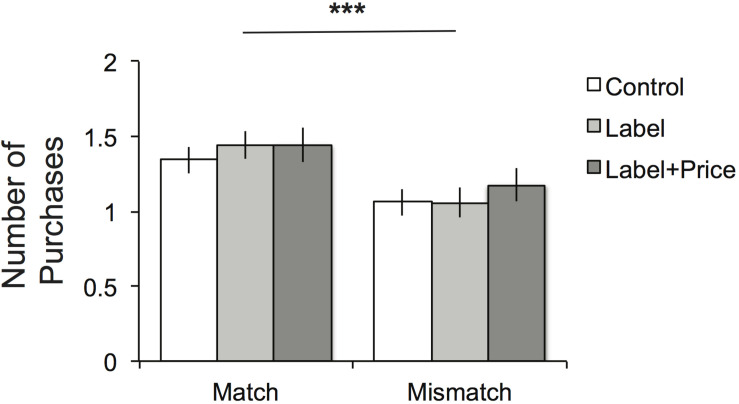
Study 2 results. Number of purchases (“yes”) decisions made in each of the six between-subject experimental conditions. ***p < 0.001.

Although this experiment measured purchase intent in a hypothetical situation, for consistency with Study 1 and improved external validity, purchase intent ratings were translated into a binary purchase decision. Thus the no answers (Strong No, No and Weak No; 1–3 on the scale) were coded as 0, and the yes answers (Weak Yes, Yes, and Strong Yes) were coded as 1. This purchase decision was modeled with a mixed effects logistic regression with dummy variables for the matched condition, label condition, and label + price condition as well as subject random effects (estimated using Stata/SE software). This demonstrated a significant effect of the matched condition (*b* = 0.386, SE = 0.120, *p* < 0.001). However, there was no significant effect of the different information presentations as indicated by the label term (*b* = −0.007, SE = 0.147, *p* = 0.961) or the label + price term (*b* = 0.101, SE = 0.146, *p* = 0.488; test for equality of label and label + price, *p* = 0.457). These effects are reflected in Figure 3, which shows the average number of products purchase in each condition. Given that these decisions were not incentivized, participants may not have been as focused on the choice as a yes/no decision. However, the same pattern of findings arises when the data was analyzed by examining the full response scale instead of a binary purchase representation^2^. Thus the display-set effect was robust to varying the amount of available information about the display items, such as their labels and prices.

We examined the effects of the various conditions on the perceived overall attractiveness of the offer using an ANOVA with repeated-measures representing the three within-subject offers (art, game and towels; estimated using SPSS software). There was a main effect of display-only item category on the perceived attractiveness of the overall offer, such that matched sets were perceived as more attractive (*M* = 4.38, SE = 0.078) compared to mismatched sets (*M* = 3.913, SE = 0.078; *F*(1,406) = 17.63, *p* < 0.001)^3^. There was no significant main effect of the three information conditions (*F*(2,406) = 0.647, *p* = 0.524) nor was there a significant interaction between category and information (*F*(2,406) = 1.27, *p* = 0.282). These results suggest the display-set effect was not impacted by including the display items’ labels and prices. In addition, the between-subjects nature of this study means that participants were rating offers that featured the same exact target products, which might have been expected to dominate the value of the offers since they were the only items under consideration in the purchase decision. However, an important limitation is that it is possible that the display items in the mismatched condition were perceived to be less valuable than the display items shown in the matched condition. In addition, these ratings were taken after the purchase choice, and thus might reflect the decision itself. Conservatively then, this data may be best described as consistent with the proposal that matched display-only items increase the attractiveness of the decision opportunity as a whole.

## Discussion

When shopping online, it is common to see information about other options displayed on product pages otherwise dedicated to facilitating the purchase of a specific target item. These display items are often generated by recommendation systems with the intent of spurring additional or subsequent buying considerations. Here, however, we replicate findings that display items can interact with and influence purchase likelihood of the target depending on whether they are matched or mismatched to the target’s category (Karmarkar, 2017; Friedman et al., 2018). We used eye-tracking to enhance our understanding of this effect by exploring how attention, as measured by fixation duration, might vary across those conditions. We provide novel evidence that neither attention to (nor valuation of) the target changes depending on the nature of the display, despite differences in purchase rates. However, there are significant differences related to the display items, consistent with a framework in which display items act in multiple ways to change the perceptions of the overall decision rather than only perceptions of the target.

We hypothesized that mismatched display items might raise awareness of opportunity costs and/or engage competing consideration of other categories (e.g., Jones et al., 1998; Frederick et al., 2009; Spiller, 2011). In line with this, (conditional on shoppers allocating a non-zero amount of attention to the display-only items) Study 1 demonstrated that they spend more time looking at the display items when the target and the display come from different (mismatched) categories than when they come from the same (matched) category. This is consistent with recent results connecting increased opportunity cost salience with decreases in purchase of a target item (Smith et al., 2019). This suggests the inclusion of otherwise neglected product categories effectively change the scope of the question being asked, broadening the focus beyond a single product category, and potentially introducing other types of attribute evaluations and associations. Notably, the increase in fixation times on mismatched items might also be more broadly described as an increased saliency compared to the matched items ones. Matched items may be more fluent or easily processed under the same conceptual schema as the target. Thus the salience of mismatched items might relate to additional engagement related to retrieval of non-target category information, estimates of value and different attributes and purchase criteria.

For matched categories, we proposed that the display items could act as reinforcing or positive evidence, increasing the category’s attractiveness and thus increasing the attractiveness of the decision as a whole. A similar prediction is made by the set-fit effect (e.g., Evers et al., 2014) in which sets that appear to “fit together” by an organizational principal are perceived to be more attractive than mismatched collections. Supporting this, Study 1 found that increases in the perceived value of the display items increase purchase likelihood for matched (but not mismatched) displays, and Study 2 showed that the overall offering (target + display) is rated as more attractive when the display items match the target category.

The design of the eye-tracking stimuli in Study 1 examined shopper attention in a simplified setting. Though our analyses focused on product images, only the target product was also accompanied by a descriptive text label and price information. It is likely that this additional information, particularly price, would be available for display items as well in commercial settings. This would suggest more competition for consumers’ attention, and raises the possibility that attention would vary in ways other than the ones tested, leading to different purchasing outcomes. To address these concerns, in Study 2 we demonstrated that the display-set effect replicates in settings involving identical available information across target and display items (e.g., text-label only, and text-label with price). Though introducing price information can bias preferences (e.g., Lee et al., 2015) our results suggest that doing so does not disrupt the display-set effect. This offers additional translational value of these findings for practical application.

One limitation to these results is that non-target items are presented as display-only, meaning that participants have no way to access or choose them. This is intended to reflect the nature of an online product-specific page, by focusing the participants’ decision-making ability on the target item. It also allows us to study the display set phenomenon in ways that are distinct from phantoms or stockouts, which are presented as attractive valid choice options that are then revealed to be unavailable (e.g., Pratkanis and Farquhar, 1992; Farquhar and Pratkanis, 1993; Fitzsimons, 2000). However, it is more rigid than a normal e-commerce experience. While it’s often true that shoppers are farther along the decision funnel by the time they arrive at a specific product page, they do retain the ability to switch over to display items’ pages, or navigate there subsequently to select multiple products for purchase. This raises an opportunity for future research about how the magnitude of display effects may depend on the relative switching costs, budget limitations, or inertia in forgoing the current offer to explore different opportunities.

Additional considerations arise from the detection of fixations and the spatial organization of the stimuli on the screen in these experiments, as illustrated in Figure 1. This design was chosen to highlight the target product in the center, to “embed” the target in the context of the display items, and also to allow for clear identification of attention (fixation) on the display vs. target images. However, an absence of observed fixations in an AOI could reflect an absence of attention, or could arise from the variance in fixation detection across participants and across trials (or as cross classification as a saccade). Thus our analyses and ensuing results are conditional on detection of at least one fixation in the relevant AOIs. In addition, there is an interesting question of whether eye movements between the target and display information (saccades) could be used to investigate differences in the qualitative type of process or comparisons made during matched and mismatched trials. The current design may interfere with interpretation of such analyses because shifts in gaze between display items involve longer distances and can be interrupted by the target. Thus from a conceptual standpoint, varying the positions of the display and target images, would be a useful addition to future studies.

This research centers on the impact of display congruency in ways that align with a frequently encountered piece of the e-commerce decision-making process: when shoppers have landed on a particular product page. As outlined in prior work, we can consider online shopping behavior leading up to this situation as either experiential or goal-directed (e.g., Hoffman and Novak, 1996; Novak et al., 2003). Our setting makes no assumptions about the consumers’ wants or needs prior to presenting the item, and is agnostic as to whether the product page was found by accident, direct or outside link. As such, our findings reflect more of an experiential or browsing process as defined by Novak et al. (2003). Thus they offer a useful complement to findings from research investigating the congruency of options in which shopping decisions are being made with a specific goal in mind (e.g., Reinholtz et al., 2015; Friedman et al., 2018). For example, Friedman et al. (2018) show that the presence of alternatives decreases purchase rates of a target item overall, but similar ones decrease purchase less than dissimilar ones. The authors lay out a goal theoretic model in which adding similar alternatives largely preserve the focal goal, but dissimilar ones diminish it by raising competing non-focal goals. In our studies, participants are not guided by an *a priori* goal, rather the likelihood of purchase is driven by endogenous preferences for the individual items and likely influenced by preference construction elicited by the immediate context (e.g., Bettman et al., 1998). Thus while the *relative* pattern of lower purchase rates arising from incongruent display items is conserved, the display-set effects suggest that matched products actually enhance purchase rates for experiential consumers.

Finally, this work offers a potentially useful parallel between a common incentive compatible study methodology and the consumer decision process online. Like many consumer neuroscience experiments, Study 1 involves a series of purchase decisions, with one choice being selected at random to “count for real” at the end. A practical purpose of this is to ensure that a single endowment can provide sufficient funds for participants to make multiple choices without raising budget constraints. However, this process can also reflect the idea of having participants select which options they “would” purchase by adding them to a shopping cart throughout the study, with checkout at the end, which does resemble the progression of e-commerce experiences. While academic studies can sometimes face challenges with external validity, this experiment structure highlights distinct opportunities for customer behavior research related to how display information impacts digital baskets that persist over site visits, wish lists, and other forms of purchase intentions that may be unique to the online experience (see also Senecal et al., 2005; Kukar-Kinney and Close, 2010; Popovich and Hamilton, 2020).

## Data Availability Statement

The datasets presented in this study can be found in online repositories. The names of the repository/repositories and accession number(s) can be found below: https://researchbox.org/304, https://osf.io/mbkf9/?view_only=a00accf08563441ca313cc1bfb73f2e3.

## Ethics Statement

The studies involving human participants were reviewed and approved by Committee on the Use of Human Subjects (Harvard University Area Institutional Review Board) Human Research Protection Programs (UCSD Institutional Review Board). Handwritten signatures for informed consent for participation were not required for this study in accordance with the national legislation and the institutional requirements.

## Author Contributions

UK formulated the research question, designed the studies, and wrote the manuscript. All authors were involved in elements of study implementation, data collection, and data analysis, and approved the submitted version.

## Conflict of Interest

SH is currently affiliated with a program run by Antler. The remaining authors declare that the research was conducted in the absence of any commercial or financial relationships that could be construed as a potential conflict of interest.

## Publisher’s Note

All claims expressed in this article are solely those of the authors and do not necessarily represent those of their affiliated organizations, or those of the publisher, the editors and the reviewers. Any product that may be evaluated in this article, or claim that may be made by its manufacturer, is not guaranteed or endorsed by the publisher.

## Footnotes

1 Regression analyses conducted on fixation times spent on the target label AOI and target price AOI similarly failed to find differences between the two display-item conditions.

2 Using SPSS software, an ANOVA with repeated-measures across the three product decisions using the full response scale revealed a significant main effect of display item category such that purchase intent was significantly higher for matched displays (*M* = 3.3, SE = 0.065) compared to mismatched displays (*M* = 2.88, SE = 0.064; *F*(1,408) = 21.104, *p* < 0.001). There was no significant effect of information condition (*F*(2,408) = 0.048, *p* = 0.953), nor was there a significant interaction effect (*F*(2,408) = 0.231, *p* = 0.794).

3 Two participants did not complete the display attractiveness ratings.

## References

[B1] Bettman J. R. Luce M. F. Payne J. W. (1998). Constructive consumer choice processes. *J. Consum. Res.* 25 187–217. 10.1086/209535

[B2] Botti S. Iyengar S. S. (2004). The psychological pleasure and pain of choosing: when people prefer choosing at the cost of subsequent outcome satisfaction. *J. Pers. Soc. Psychol.* 87 312–326. 10.1037/0022-3514.87.3.312 15382982

[B3] Doyle J. R. O’Connor D. J. Reynolds G. M. Bottomley P. A. (1999). The robustness of the asymmetrically dominated effect: buying frames, phantom alternatives, and in-store purchases. *Psychol. Mark.* 16 225–243. 10.1002/(sici)1520-6793(199905)16:3<225::aid-mar3>3.0.co;2-x

[B4] Evers E. R. Inbar Y. Zeelenberg M. (2014). Set-fit effects in choice. *J. Exp. Psychol. Gen.* 143 504–509. 10.1037/a0033343 23773159

[B5] Farquhar P. H. Pratkanis A. R. (1993). Decision structuring with phantom alternatives. *Manage. Sci.* 39 1214–1226. 10.1287/mnsc.39.10.1214 19642375

[B6] Fitzsimons G. J. (2000). Consumer Response to Stockouts. *J. Consum. Res.* 27 249–266. 10.1086/314323

[B7] Frederick S. Novemsky N. Wang J. Dhar R. Nowlis S. (2009). Opportunity cost neglect. *J. Consum. Res.* 36 553–561. 10.1086/599764

[B8] Friedman E. M. Savary J. Dhar R. (2018). Apples, oranges, and erasers: the effect of considering similar versus dissimilar alternatives on purchase decisions. *J. Consum. Res.* 45 725–742. 10.1093/jcr/ucy023

[B9] Gourville J. T. Norton M. I. (2014). *Consumer behavior and the buying process.” HBS no. 8167.* Boston: Harvard Business School Publishing.

[B10] Greenberg A. E. Spiller S. A. (2016). Opportunity cost neglect attenuates the effect of choices on preferences. *Psychol. Sci.* 27 103–113. 10.1177/0956797615608267 26573905

[B11] Häubl G. Trifts V. (2000). Consumer decision making in online shopping environments: The effects of interactive decision aids. *Mark. Sci.* 19 4–21. 10.1287/mksc.19.1.4.15178 19642375

[B12] Hoffman D. L. Novak T. P. (1996). Marketing in hypermedia computer-mediated environments: conceptual foundations. *J. Market.* 60 50–68. 10.2307/1251841

[B13] Huber J. Payne J. W. Puto C. (1982). Adding asymmetrically dominated alternatives: violations of regularity and the similarity hypothesis. *J. Consum. Res.* 9 90–98. 10.1086/208899

[B14] Jones S. K. Frisch D. Yurak T. J. Kim E. (1998). Choices and opportunities: Another effect of framing on decisions. *J. Behav. Decis. Mak.* 11 211–226. 10.1002/(sici)1099-0771(199809)11:3<211::aid-bdm298>3.0.co;2-o

[B15] Karmarkar U. R. (2017). The impact of “display-set” options on decision-making. *J. Behav. Decis. Mak.* 30 744–753. 10.1002/bdm.1998

[B16] Karmarkar U. R. Shiv B. Knutson B. (2015). Cost conscious? The neural and behavioral impact of price primacy on decision making. *J. Market. Res.* 52 467–481. 10.1509/jmr.13.0488 11670861

[B17] Knutson B. Rick S. Wimmer G. E. Prelec D. Loewenstein G. (2007). Neural predictors of purchases. *Neuron* 53 147–156. 10.1016/j.neuron.2006.11.010 17196537 PMC1876732

[B18] Krajbich I. Armel C. Rangel A. (2010). Visual fixations and the computation and comparison of value in simple choice. *Nat. Neurosci.* 13 1292–1298. 10.1038/nn.2635 20835253

[B19] Krajbich I. Lu D. Camerer C. Rangel A. (2012). The attentional drift-diffusion model extends to simple purchasing decisions. *Front. Psychol.* 3:193. 10.3389/fpsyg.2012.00193 22707945 PMC3374478

[B20] Kukar-Kinney M. Close A. G. (2010). The determinants of consumers’ online shopping cart abandonment. *J. Acad. Mark. Sci.* 38 240–250. 10.1007/s11747-009-0141-5

[B21] Lebreton M. Jorge S. Michel V. Thirion B. Pessiglione M. (2009). An automatic valuation system in the human brain: evidence from functional neuroimaging. *Neuron* 64 431–439. 10.1016/j.neuron.2009.09.040 19914190

[B22] Lee L. Lee M. P. Bertini M. Zauberman G. Ariely D. (2015). Money, time, and the stability of consumer preferences. *J. Mark. Res.* 52 184–199. 10.1509/jmr.10.0386 11670861

[B23] Levy I. Lazzaro S. C. Rutledge R. B. Glimcher P. W. (2011). Choice from non-choice: predicting consumer preferences from blood oxygenation level-dependent signals obtained during passive viewing. *J. Neurosci.* 31 118–125. 10.1523/jneurosci.3214-10.2011 21209196 PMC3078717

[B24] Louie K. Khaw M. W. Glimcher P. W. (2013). Normalization is a general neural mechanism for context-dependent decision making. *Proc. Natl. Acad. Sci. U. S. A.* 110 6139–6144. 10.1073/pnas.1217854110 23530203 PMC3625302

[B25] Northcraft G. B. Neale M. A. (1986). Opportunity costs and the framing of resource allocation decisions. *Organ. Behav. Hum. Decis. Process.* 37 348–356. 10.1016/0749-5978(86)90034-8

[B26] Novak T. P. Hoffman D. L. Duhachek A. (2003). The influence of goal−directed and experiential activities on online flow experiences. *J. Consum. Psychol.* 13 3–16. 10.1207/S15327663JCP13-1&2_01

[B27] Pettibone J. C. Wedell D. H. (2000). Examining models of nondominated decoy effects across judgment and choice. *Organ. Behav. Hum. Decis. Process.* 81 300–328. 10.1006/obhd.1999.2880 10706818

[B28] Popovich D. Hamilton R. (2020). Intermediate Choice Lists: How Product Attributes Influence Purchase Likelihood in a Self-Imposed Delay. *J. Retail.* 97 251–266. 10.1016/j.jretai.2020.07.002

[B29] Pratkanis A. R. Farquhar P. H. (1992). A brief history of research on phantom alternatives: Evidence for seven empirical generalizations about phantoms. *Basic Appl. Soc. Psychol.* 13 103–122. 10.1207/s15324834basp1301_9

[B30] Prelec D. Loewenstein G. (1998). The red and the black: Mental accounting of savings and debt. *Market. Sci.* 17 4–28. 10.1287/mksc.17.1.4 19642375

[B31] Reinholtz N. Bartels D. M. Parker J. R. (2015). On the mental accounting of restricted-use funds: How gift cards change what people purchase. *J. Consum. Res.* 42 596–614.

[B32] Scarpi D. Pizzi G. (2013). The impact of phantom decoys on choices and perceptions. *J. Behav. Decis. Mak.* 26 451–461. 10.1002/bdm.1778

[B33] Senecal S. Kalczynski P. J. Nantel J. (2005). Consumers’ decision-making process and their online shopping behavior: a clickstream analysis. *J. Bus. Res.* 58 1599–1608. 10.1016/j.jbusres.2004.06.003

[B34] Shenhav A. Karmarkar U. R. (2019). Dissociable components of the reward circuit are involved in appraisal versus choice. *Sci. Rep.* 9 1–12. 30760824 10.1038/s41598-019-38927-7 PMC6374444

[B35] Simonson I. (1989). Choice based on reasons: the case of attraction and compromise effects. *J. Consum. Res.* 16 158–174. 10.1086/209205

[B36] Smith A. Bernheim B. D. Camerer C. Rangel A. (2014). Neural activity reveals preferences without choices. *Am. Econ. J. Microecon.* 6 1–36. 10.1257/mic.6.2.1 25729468 PMC4339868

[B37] Smith S. Spiller S. A. Krajbich I. (2019). “The Role of Attention in Opportunity Cost Neglect,” in *NA - Advances in Consumer Research Volume 47*, eds Bagchi R. Block L. Lee L.. (Duluth, MN: Association for Consumer Research). 281–285

[B38] Spiller S. A. (2011). Opportunity cost consideration. *J. Consum. Res.* 38 595–610. 10.1086/660045

[B39] Thaler R. (1985). Mental accounting and consumer choice. *Mark. Sci.* 4 199–214. 10.1287/mksc.4.3.199 19642375

[B40] Trueblood J. S. Pettibone J. C. (2017). The phantom decoy effect in perceptual decision making. *J. Behav. Decis. Mak.* 30 157–167. 10.1002/bdm.1930

[B41] Tusche A. Bode S. Haynes J. D. (2010). Neural responses to unattended products predict later consumer choices. *J. Neurosci.* 30 8024–8031. 10.1523/jneurosci.0064-10.2010 20534850 PMC6632699

[B42] Webb R. Glimcher P. W. Louie K. (2021). The normalization of consumer valuations: Context-dependent preferences from neurobiological constraints. *Manage. Sci.* 67 93–125. 10.1287/mnsc.2019.3536 19642375

