# Supplementary material for: Category Congruence of Display-Only Products Influences Attention and Purchase Decisions
Source: Front Neurosci. 2021 Aug 26;15:610060. doi: 10.3389/fnins.2021.610060 (PMC8427019; doi:10.3389/fnins.2021.610060)
Supplement: Supplementary file 1 [file Data_Sheet_1.pdf]

Supplemental Materials

Study 1 Methods : Stimulus Design

The display set effect relating to purchase differences dependent on matched or mismatched display items been demonstrated across a wide range of consumer product categories (Karmarkar 2017, Friedman, Savary and Dhar, 2018). These studies have shown that the effects do not not appear to be moderated by factors such as hedonic or utilitarian purposes, consumable versus durable goods, or variations in non-choice image sizes, shapes and colors.

Along these lines, the research design was aimed at measuring condition-specific effects separately from the nature or attributes of any particular target item or its category. Thirty-six popular retail products aimed at a demographic consistent with our 18-40 year old sample were selected as target items. Each was given a number (1-36) and then assigned to one of three "sublists" of items (list A, list B, list C) such that product categories were distributed among the lists. For example, there were two DVDs selected to be target products, and these were separated into different sub-lists.

The overall presentation order of all 36 items was pseudorandomized. This was done by first creating a randomized sequence without replacement from the integers 1-36 (https://www.random.org/integers/) and then ordering the target products based on that randomization. The resulting sequence was revised to avoid any "runs" of more than 2 items were re-ordered. For example, a run of three A items (AAA) would have been reconfigured to AAB. Finally, it was confirmed that the list did not create sequential decisions involving similar target categories. For example, the two DVD decisions are separated by at least one choice about a product from a different category.

All participants made their decisions about this list of targets in the same order. However, there were three versions of the task that counterbalanced the display conditions assigned to items from list A, B and C. In V1, all items from list A were landscape, from list B were matched and list C was mismatched. In V2, A was mismatched, B was landscape, C was matched. And of course in V3, A was matched, B was mismatched and C was landscape. Thus while the items were presented in an identical sequence for all participants, the order of the conditions (matched, mismatched and landscape) varied across participants.

To create the displays, for each of the 36 targets, two similar display items that were listed in the same product category on at least one major online retailer were selected. For example, if the target was a Harvard scarf, then Harvard gloves and a Harvard winter hat were selected as the matching display items.

For any of the three versions of the study, the sublist assigned to the matched condition had its targets presented with these two matched display items. The sublist assigned to the landscape condition had its targets presented with a pair of landscape images (e.g. a Harvard scarf was presented with two landscape pictures). The sublist assigned to the

mismatched condition had its targets presented with the two display items that “matched” with the targets in the landscape sublist (e.g. a Nalgene water bottle was presented with the Harvard scarf and Harvard winter hat.)

This meant that display items viewed with a matched target in one of the task versions would be viewed as mismatched in one of the other (counterbalanced) versions. This design was intended to maximize the importance of the congruency of the display rather than the specific values of the individual display items.

The 36 target products and their sub-lists are represented in the table below, and full trial information for each participant can be accessed in the data repository for the paper: https://researchbox.org/304. Full individual eyetracking data for these studies can be viewed and/or downloaded at the following OSF link : https://osf.io/mbkf9/?view_only=a00accf08563441ca313cc1bfb73f2e3.

Target Product	Trial	Sub-list
Moleskine Classic Notebook	1	A
Planet Earth : Volume 1 DVD	2	C
Nano Wireless Travel Mouse	3	C
Cuisinart Mini Food Processor	4	B
Pilot Rolling Ball "BeGreen" Pens (12 ct.)	5	C
Dryel Home Dry-Cleaning Kit	6	2
Mr. Coffee Programmable CoffeeMaker	7	A
Brita Water Filter Pitcher	8	C
Cards Against Humanity Game	9	B
House of Cards Season 1 DVD	10	A
Nalgene Triton BPA-Free Water Bottle	11	B
Travel Neck Pillow	12	A
Com-Pad Portable Phone Charger	13	A
Bonsai Tree Kit	14	C
Ghirardelli Assorted Chocolates	15	B
Dorcy Waterproof LED Flashlight	16	C
National Geographic 1 Year Subscription	17	B
Bormioli Murano Glasses (set of 4)	18	A
Eagle Creek Pack-It Clean/Dirty Cube	19	B
iHome Portable Speaker	20	B
Belgian Waffle Maker	21	C
Harvard Logo T-Shirt	22	C
Sony Stereo Headphones	23	A
Bike U-Lock	24	A
OralB Precision Clean Battery Toothbrush	25	C
Timex Water Resistant Sports Watch	26	C
WoodWick Vanilla Candle	27	B
LED Dimmable Desk Lamp	28	B
Pivot Flexible Powerstrip	29	C
Crest 3D Whitestrips	30	A
Venice Palazza Da Mula: Monet, Art Print	31	A
Chalkboard Mural	32	B
KIND Snacks Minis Variety Box	33	A
Game of Thrones Season 1 DVD	34	B
Bananagrams	35	C
Harvard Logo Scarf	36	A
